# A Systematic Review and Meta-Analysis of the Functional MRI Investigation of Motor Neuron Disease

**DOI:** 10.3389/fneur.2015.00246

**Published:** 2015-11-24

**Authors:** Dongchao Shen, Liying Cui, Bo Cui, Jia Fang, Dawei Li, Junfang Ma

**Affiliations:** ^1^Department of Neurology, Peking Union Medical College Hospital, Chinese Academy of Medical Sciences, Peking Union Medical College, Beijing, China; ^2^Neuroscience Center, Chinese Academy of Medical Sciences, Beijing, China

**Keywords:** motor neuron disease, amyotrophic lateral sclerosis, fMRI, systematic review, meta-analysis

## Abstract

**Background:**

To assess the use of functional magnetic resonance imaging (fMRI) in motor neuron disease (MND), a systematic review and voxelwise meta-analysis of studies comparing brain activity in patients with MND and in healthy controls was conducted to identify common findings across studies.

**Methods:**

A search for related papers published in English and Chinese was performed in Ovid Medline, Pubmed, and Embase database. Voxelwise meta-analysis was performed using signed differential mapping.

**Results:**

The findings from 55 fMRI studies on MND were tabulated, and some common findings were discussed in further details.

**Conclusion:**

These findings are preliminary, sometimes even contradictory, and do not allow a complete understanding of the functional alterations in MND. However, we documented reliable findings that MND is not confined to the motor system, but is a multisystem disorder involving extra-motor cortex areas, causing cognitive dysfunction and deficits in socioemotional and sensory processing pathways.

## Introduction

Motor neuron disease (MND) is a progressive neurodegenerative disorder primarily involving the motor neurons in the cerebral cortex, brain stem, and spinal cord. Amyotrophic lateral sclerosis (ALS) is the most common form, characterized by involvement of both upper motor neuron (UMN) and lower motor neuron (LMN), with a median survival time of 2–4 years from onset of symptoms in population-based studies (1). Other phenotypes of MND include primary lateral sclerosis (PLS, UMN affected only), progressive muscular atrophy (PMA, LMN affected only), progressive bulbar palsy (PBP, motor nuclei of pons and medulla affected mainly) and some special forms, such as flail arm or flail leg (2). The cause of MND remains largely unknown, but there is an increasing awareness that the neurodegeneration of MND is not only restricted to the motor system but also involves sensory, language, behavior and other cognitive fields. In fact, up to 50% of patients with ALS have cognitive deficits, ranging from mild cognitive impairment to overt frontotemporal dementia (FTD) (3).

Functional magnetic resonance imaging (fMRI) has been widely used to study functional changes of the brain in many neurological and psychiatric disorders including MND, since it provides high-resolution, non-invasive estimates of neural activity. The most common approach in fMRI, blood-oxygenation-level-dependent (BOLD) MRI is based on the assumption that when a neural event occurs, extraction fraction of oxygen from the local microvasculature leads to a decreased of deoxygenated hemoglobin in the activated region. Changes from diamagnetic oxyhemoglobin to paramagnetic deoxyhemoglobin that take place with the neural event leads to an alteration in T2*-weighted MRI signals. Acquisition of BOLD signals can be combined with specific tasks to perform task-associated fMRI studies, and background patterns of regional activity in the “resting state” can be studied, which is called the resting state-fMRI (RS-fMRI). The broad availability of fMRI with a multitude of motor and extra-motor paradigms has contributed to a great increase in scientific publications on functional studies in MND over the last decade (4).

Understanding patterns of functional activity in patients with MND could provide important insights on the pathophysiology of this fatal disease, as well as potential targets for therapeutic intervention. An independent meta-analysis is a powerful strategy to combine and summarize the data of interest and potentially offers insights that are not apparent from the individual studies. We, therefore, conducted a systematic review and voxelwise meta-analysis of studies comparing brain activity in patients with MND and in healthy controls (HCs) to identify common findings across studies.

## Materials and Methods

### Literature Search Methods

Ovid Medline, Pubmed, and Emabase databases were searched for studies published up to April 2015 that reported functional MRI data in patients with MND. Search terms included “motor neuron disease,” “MND,” “amyotrophic lateral sclerosis,” “ALS,” and these terms were combined using the AND operator with “functional magnetic resonance imaging,” “functional MRI,” “fMRI,” “blood oxygenation level dependent,” “BOLD,” “resting state,” and “connectivity.” Both text word and MeSH subject headings were used. Language was confined to English or Chinese, and reviews were excluded in the advanced search. The search strategy was supplemented by inspecting the reference lists of included articles.

### Inclusion Criteria

To be included in the systematic review, studies had to meet the following criteria: (1) observational study conducted in patients with MND, (2) comparison group of HCs, and (3) subjects were evaluated by fMRI. Articles were excluded if they were case reports or non-human subjects were involved. When the same study population was reported in more than one article, the data were included only once. Literature was evaluated by two independent researchers at the same time, and conflicts were resolved by a third party after discussion of each article.

### Data Extraction

For each study, following data for participants were extracted: mean age, mean disease duration, and mean revised ALS functional rating scale (ALSFRS-R) scores. Details of the paradigm of task-associated fMRI were recorded, which were divided into motor tasks (including movement execution, imagery or observation) and extra-motor tasks. The analysis methods of RS-fMRI and combined imaging techniques, such as diffusion tensor imaging (DTI) or voxel-based morphometry (VBM), were also extracted. Our major concerns were the activation or connectivity changes of MND patients compared with HCs.

### Voxelwise Meta-Analysis

Voxelwise meta-analysis was performed using the signed differential mapping (SDM) software (version 4.31, http://www.sdmproject.com/), which combines various positive features of earlier methods, such as activation likelihood estimation and multilevel kernel density analysis (5). The SDM methods have been described in details elsewhere (6, 7). Articles were selected for voxelwise meta-analysis if (1) voxel-based comparisons were made at the whole-brain level between patients with MND and HCs and (2) differences in voxel signal intensity between patients and controls were reported in Talairach or Montreal Neurological Institute (MNI) space, and studies were excluded if they used seed-based or region of interest (ROI)-based correlation analysis. The MND vs. HC contrasts to different stimuli or at rest were chosen as individual SDM analyses: (1) MND vs. HC to motor stimuli, (2) MND vs. HC to extra-motor stimuli, and (3) MND vs. HC at rest. Two researchers independently extracted activation foci from included studies, and a disagreement was ruled by a third party after discussions. The full width at half maximum (FWHM) was set at 20 mm, which had excellent control for false positives according to previous studies; and the statistical threshold was set to be a *P*-value <0.005 without correction for false discovery rate (FDR), which was found to be able to optimize the balance between sensitivity and specificity (6). Mean analysis and Jackknife sensitivity analysis were carried out. The last analysis was a meta-regression of voxel values across the studies by the ALSFRS-R scores of the patients’ samples.

## Results

### Included Papers

**Figure 1:**
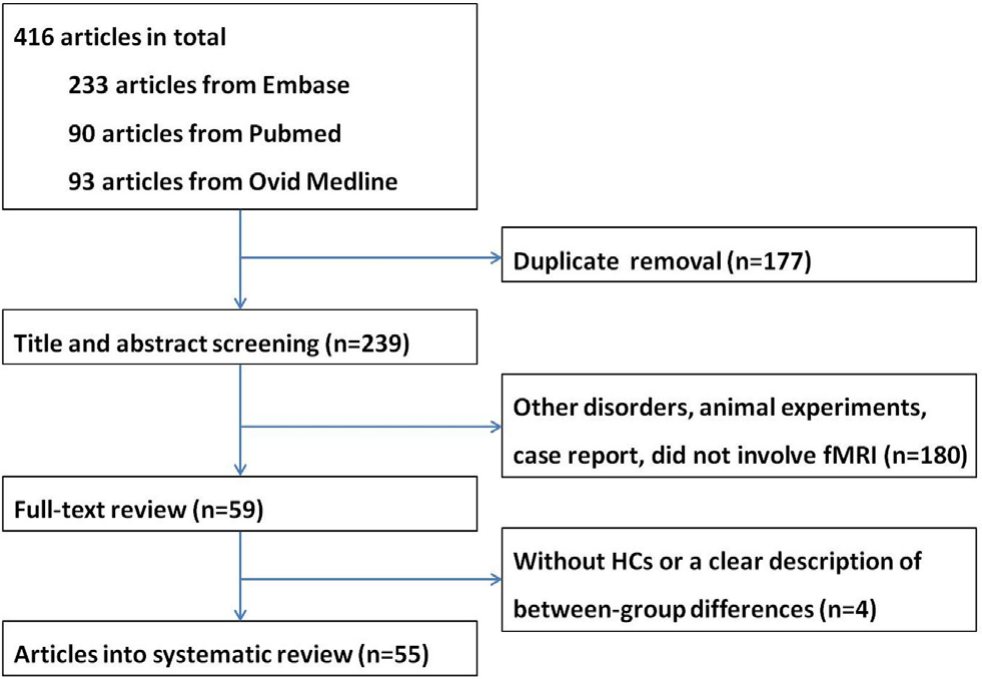
**The flow chart of the literature search in the systematic review**.

A total of 416 articles were retrieved. After removal of duplicate entries, 239 articles remained and then underwent screening according to title and abstract, resulting 180 being excluded because (1) the study focused on disorders other than MND, or (2) the study is an animal experiment or a case report, or (3) the study did not involve fMRI. After full-text review, another four papers were excluded due to lack of a HC group or a clear description of between-group differences. Eventually a total of 55 papers were included in the systematic review, including 18 task-associated fMRI studies using motor paradigms (8–25), 12 studies using extra-motor paradigms (26–37) and 25 RS-fMRI studies (38–62) (Tables 1–3). Twelve research groups (8, 12, 14, 16, 17, 26, 27, 39, 40, 42, 43, 49) reported more than one article that we could not identify whether their data were derived from the same patients sample or not; however, because different tasks or analytical methods were used, results of all of these articles are presented in the systematic review. A flow chart of publication selection is presented in Figure 1.

**Table 1:** **Task-associated fMRI studies in MND patients using motor paradigms**.

Study	Subjects (mean age, years)	Disease duration (months)	ALSFRS-R score	Task design	Main findings in MND patients compared to HCs	Other findings
Konrad (8)	11 ALS (33), 13 HCs (44)	35	NA	Block design: finger flexion with dominant hand	Motor cortex activation located more anteriorly; increased volumes of activation in SMA and cingulate motor areas, contralateral inferior lateral PMC, bilateral parietal cortex	–
Schoenfeld (9)	6 ALS (56.2), 6 HCs (57.1)	16	40	Block design: consecutive button presses with right hand, fixation/rest as baseline	1. More activated in bilateral motor areas and PMC; additional activated in bilateral cerebellar areas. 2. Less activated in contralateral M1	–
Konrad (10)	10 ALS (44), 10 HCs (45)	21.5 (since diagnosis)	NA	Block design: finger flexion with right hand, rest as baseline	More active in right cerebellar hemisphere, right basal ganglia (especially putamen), bilateral brainstem (especially dorsal pons), right SMA; additional activated in right cingulate areas, bilateral inferior PMC, bilateral basal ganglia, left cerebellar hemisphere	–
Han (11)	15 ALS (51.3), 15 HCs (49.5)	13.1	NA	Block design: sequential finger tapping with right and left hands, rest as baseline	Activation larger in bilateral primary sensorimotor cortex, bilateral PMC, bilateral SMA, ipsilateral cerebellum; extra activation in ipsilataral inferior lateral PMC, bilateral posterior limb of internal capsule, contralateral cerebellum	–
Tessitore (12)	16 ALS (53.9), 13 HCs (54)	39.8	27.4	Block design: visually paced button press with right hand, rest as baseline	1. Recruited more left anterior putamen. 2. Less activity in left M1, SMA, right posterior parietal cortex	Patients with greater UMN involvement had more robust activation of ACC and right caudate nucleus than patients with greater LMN involvement
Lule (13)	14 ALS (53), 15 HCs (55)	40	33.5	Block design: grip force task and imagery of the same movement with right, left or both hands, rest as baseline	1. Stronger response within M1 and PMC for imagery and execution. 2. Differences persisted 6 months later with additional activity in precentral gyrus and frontoparietal network for motor imagery, increased with impairment	–
Stanton (14, 15)	16 ALS (55.1), 9 peripheral lesions (51.9), 17 HCs (55.3)	25.9	41.1	Block design: moving a joystick with right hand and imagery of the same movement, rest as baseline	1. Execution: increased activation in primary sensorimotor cortex and extended posteriorly into inferior parietal lobule and inferiorly into superior temporal gyrus, reduced activation in left DLPFC and extended into anterior and medial prefrontal cortex and SMA. 2. Imagery: reduced activation in left inferior parietal lobule, ACC and medial prefrontal cortex	–
Li (16)	10 ALS (45.8), 10 HCs (age matched)	21.4	38.4	Event-related design: voluntary saliva swallow, rest as baseline	1. For patients without dysphagia, increased activation in bilateral postcentral gyrus. 2 For patients with dysphagia, reduced activation in bilateral postcentral gyrus	1. ALS patients without dysphagia showed increased activity in bilateral precentral and postcentral gyri than patients with dysphagia, with additional activity in left thalamus. 2. Cerebral activation map changes correspond to diffusion abnormalities by DTI in ALS
Mohammadi (17)	22 ALS (57), 5 Kennedy syndrome (59), 22 HCs (61)	14	39.5	Block design: tongue vertical movements, rest as baseline	For patients with bulbar sign, decrease of cortical activity (pre- and postcentral areas) and missing thalamic activity	–
Mohammadi (18)	22 ALS (57), 22 HCs (61)	14	39.5	Block design: flexion and extension of fingers in right hand, rest as baseline	For patients with MRC-Megascores of 5, increased activation in bilateral M1, S1 and posterior PMC, contralateral putamen, bilateral thalamus, SMA	Movement related signal change and beta weights extracted from the activated cluster were unchanged relative to controls in patients with no weakness, but a marked decrease in patients with weakness
Kollewe (19)	20 ALS (59), 20 HCs (52)	NA	38.5	Block design: tongue vertical movements, flexion and extension of fingers in right hand, rest as baseline	1. During hand movement, increased activity in bilateral M1, S1, posterior PMC and SMA. 2. For tongue movement of patients with bulbar sign, decreased activity in M1, S1 and posterior PMC	ALSFRS-R score was positively correlated with signal change in hand area during hand movements and in tongue area during tongue movements
Heimrath (20)	7 ALS (not given), 14 HCs (not given)	NA	NA	Block design: movement imagery and visual perception including 4 isolated movements, 4 body related movements, 4 movements which can be performed also in later stage ALS, and 1 control movement	1. During movement perception, more activity in areas for higher order movement representation (e.g., BA 40), less activity in right PMC. 2. During movement imagery, more activity in PMC, less activity in subcortical (e.g., putamen) and cortical (e.g., hippocampus) structures related to motor memory	More advanced disease corresponded to stronger activity in areas of higher order movement representation (e.g., BA 40)
Cosottini (21)	20 ALS (58), 16 HCs (50.6)	20.1	38.2	Block design: handgrip motor task with right, left or both hands simultaneously, rest as baseline	1. Enhanced activation in ventral premotor frontal areas and parietal cortex, prevalent in left. 2. Hypoactivation in primary sensory motor cortex and frontal dorsal PMC	1. Activation in frontoparietal motor circuit paralleled with disease progression rate. 2. Cerebral activation changes corresponded to cortical regions of atrophy by VBM
Poujois (22)	19 ALS (63.8), 21 HCs (60.3)	18.2	35.3	Block design: execution or imagery of opening and closing right or left hand, rest as baseline	1. During execution of right-hand movement, higher activity in left M1, bilateral S1 and parietal cortices (including precuneus). 2. During Imagery of right-hand movement, increased activity in left M1 and S1. 3. No difference was found for left-hand tasks	Controlateral parietal activity was inversely correlated with disease progression and ipsilateral S1 activations with the severity of the right-arm deficit
Flanagan (23)	22 ALS (not given), 18 HCs (age matched)	NA	NA	Block design: action observations involved hand-object interactions and no interaction as control condition	Reduced activation in right dorsal, ventral PMC and inferior frontal gyrus	–
Li (24)	30 ALS (53.5), 30 HCs (51.7)	24.5	36.5	Block design: watch a videotape showing repetitive flexion-extension of fingers in right hand, rest as baseline	1. Greater activation in bilateral dorsal lateral PMC, inferior parietal gyrus and SMA. 2. Greater activation in M1 and dorsal lateral PMC areas related to movement rate. 3. Greater activation in bilateral superior parietal gyrus and right inferior frontal gyrus related to movement complexity	–
Jelsone-Swain (25)	19 ALS (57.2), 18 HCs (59.9)	47	36.8	Block design: 1.Action observation and execution (squeezing a ball), rest as baseline. 2. Action understanding	1. During action-execution and observation, greater activity in right inferior operculum, PMC and M1, left inferior parietal lobe. 2. During action understanding, greater activity in right inferior occipital gyrus, reduced activity in right prefrontal cortex including triangularis, bilateral orbital regions, bilateral temporal lobe and occipital lobe	–

*ACC, anterior cingulate cortex; ALS, amyotrophic lateral sclerosis; BA, Brodmann; DLPFC, dorsolateral prefrontal cortex; DTI, diffusion tensor imaging; fMRI, functional MRI; HC, healthy control; LMN, lower motor neuron; M1, primary motor cortex; MND, motor neuron disease; MRC, medical research council; NA, not available; PMC, premotor cortex; S1, primary sensor cortex; SMA, supplementary motor area; UMN, upper motor neuron; VBM, voxel-based morphometry*.

**Table 2:** **Task-associated fMRI studies in MND patients using extra-motor paradigms**.

Study	Subjects (mean age, years)	Disease duration (months)	ALSFRS-R score	Task design	Main findings in MND patients compared to HCs	Other findings
Abrahams (26)	28 ALS (57.3), 18 HCs (55)	21	NA	Block design: letter fluency and confrontation naming, say the word “rest” as baseline	1. Letter fluency: increased activation in left superior frontal gyrus, right middle temporal gyrus and inferior frontal gyrus, reduced activation in left middle temporal gyrus, precuneus, inferior frontal gyrus and inferior parietal lobe, right ACC, bilateral middle frontal gyrus. 2. Confrontation naming: increased activation in right fusiform gyrus, impaired activation in left middle temporal gyrus, middle occipital gyrus and superior temporal gyrus, right cingulate gyrus, bilateral inferior frontal gyrus and cuneus	–
Lule (27)	13 ALS (57.5), 6 tetraplegia (not given), 15 HCs (54.4)	23	36.8	Event-related design and block design: receive socioemotional stimuli from the International Affective Picture System at inclusion and 6 months later	1. Larger activity in right supramarginal area. 2. Lower activation in extrastriate visual areas	Within the ALS patients’ group a reduction of brain responses in anterior insula at the follow-up was correlated with the subjective arousal
Jawaid (28)	18 ALS and HCs (not given)	NA	NA	NA: a socioeconomic game called the “Trust task,” patients as trustees	1. Higher activity of cingulate compared to disease-free investors. 2. Lower activity of cingulated compared to disease-free trustees	–
Palmieri (29)	9 ALS (51.7), 10 HCs (51.1)	24 (since diagnosis)	37.9	Block design: emotional attribution task asked subjects to select one of three unpleasant or neutral words, memory recognition task asked subjects to recognize words presented during previous task	A general increase in activation of left hemisphere, and reduced activation in right hemisphere in both emotional tasks	–
Lule (30)	14 ALS (52.6), 18 HCs (59.6)	28	33.4	Event-related design: receive visual, auditory and somatosensory stimuli	1. Auditory stimulation: increased activity in bilateral caudate nucleus and middle frontal gyrus, lower activity in bilateral inferior frontal gyri and right cerebellum. 2. Visual stimulation: lower activity in right occipital lobe. 3. Somatosensory stimulation: no evident difference was found	Several areas with increasing/decreasing activity during different stimuli associated with physical function loss
Goldstein (31)	14 ALS (52.6), 8 HCs (52.4)	NA	NA	Block design: Stroop and negative priming tasks	1. Stroop effect: increased activation in middle temporal gyrus, superior temporal gyrus, ACC, fusiform and lingual gyri, medial frontal gyrus, inferior parietal cortex, hippocampus, caudate nucleus, insula, cerebellum (all on left). 2. Negative priming effect: reduced activation in left cingulate gyrus, precentral gyrus and medial frontal gyrus, brainstem, lingual and fusiform gyrus, cerebellum	–
Meier (32)	2 ALS (not given), 15 HCs (not given)	NA	NA	Event-related design: reward, punishment and affective-shift trials of the reversal learning task relative to a matched affectively neutral baseline	The orbitofrontal activity of case of mild impairment on neuropsychological tests sensitive to orbitofrontal cortex function and behavioral disturbance was more bilateral and more spatially extensive than controls	–
Passamonti (33)	11 ALS (45.4), 12 HCs (40.3)	19	32.1	Block design: subjects were asked to identify the emotional faces as the “target” one via a 2-choice button box	1. Emotional vs. neutral stimuli: greater responses in ventral ACC, dorsal ACC and bilateral DLPFC. 2. Altered left amygdala-prefrontal cortex connectivity. 3. Anxiety modulated right amygdale- prefrontal cortex connectivity in HCs but not in ALS patients	Reduced right PMC activity and altered left amygdale-SMA connectivity were associated with longer disease duration and greater disease severity
Witiuk (34)	12 ALS (61.6), 12 HCs (61.6)	37.3 (since diagnosis)	36.3	Event-related design: 1.anticatch trials, 2.procatch trials, 3.correct antisaccade trials, 4.correct prosaccade trials, 5. corrected antisaccade direction errors, 6.invalid trials, fixation trials as baseline	1. Increased activation in supplementary eye fields and frontal eye fields. 2. Reductions in DLPFC activation	The ALS group showed reduced saccadic latencies that correlated with increased activation across the oculomotor saccade system
Mohammadi (35)	17 ALS (not given), 17 HCs (age matched)	NA	NA	Block design: go-stop task	1. Stronger inhibition-related activity in inferior, superior and middle frontal gyrus, putamen and pallidum, Stronger execution-related activity in contralateral sensorimotor cortex. 2. Weaker error-related activity in bilateral insula	–
Stoppel (36)	14 ALS (60.3), 14 HCs (59.7)	18.3	38.2	Block design: a modified go/no-go task at inclusion and 3 months later	1. Patients’ motor activations were higher during the initial measurement, and declined during the 3-month interval. 2. Novelty-evoked hippocampal activity increased across 3 months	1. There was a positive correlation between the ALSFRS-R or MRC-Megascores and the decline in motor activity, but a negative one with the hippocampal activation-increase. 2. There was a close overlap between functional alterations and structural changes by VBM
Raaphorst (37)	21 ALS (60.3), 18 PMA (60.4), 17 HCs (59)	22.2 (ALS), 26.0 (PMA)	40.0 (ALS), 41.5 (PMA)	Block design: letter and category fluency tasks, counting backward as baseline	Letter fluency: 1.For patients with PMA, lower activation in left inferior frontal gyrus and ACC; 2. For patients with ALS, lower activation in left inferior frontal gyrus and middle frontal gyrus	–

*ACC, anterior cingulate cortex; ALS, amyotrophic lateral sclerosis; ALSFRS-R, revised ALS functional rating scale; DLPFC, dorsolateral prefrontal cortex; fMRI, functional MRI; HC, healthy control; MND, motor neuron disease; MRC, medical research council; NA, not available; PMA, progressive muscular atrophy; PMC, premotor cortex; SMA, supplementary motor area; VBM, voxel-based morphometry*.

**Table 3:** **RS-fMRI studies in MND patients**.

Study	Subjects (mean age, years)	Disease duration (months)	ALSFRS-R score	Analysis methods	Main findings in MND patients compared to HCs	Other findings
Mohammadi (38)	20 ALS (55), 9 LMN affection (58), 20 HCs (57)	14	40	ICA	1. DMN: less activation in ventral ACC, PCC and bilateral inferior parietal cortex. 2. SMN: less activation in PMC	–
Jelsone-Swain (39)	20 ALS (58.3), 20 HCs (57.5)	17.3	39.6	Graph theory	1. Overall systemic decrease in FC between right and left motor cortices in patients with limb-onset. 2. Pronounced disconnection between dorsal ROI pairs of M1	Dorsal ROI connectivity strength was negatively correlated with hand strength disparity
Verstraete (40)	12 ALS (48.7), 12 HCs (49.6)	14.3	39.5	Graph theory, combined with DTI and SBM	Overall functional organization of motor network was unchanged	The FC level of motor network was correlated with disease progression rate in ALS patients
Filippi (41)	18 ALS (not given), 15 HCs (not given)	NA	NA	ICA, combined with VBM	1. Dysfunction of resting state connectivity of SMN. 2. Decreased average percentage signal change of resting state fluctuations in bilateral primary sensorimotor cortex and cerebellum, SMA, left inferior frontal gyrus and inferior parietal lobule	–
Douaud (42)	25 ALS (59), 15 HCs (53)	44	34	Tractography-derived FC analysis, combined with DTI	Increase of FC in primary sensorimotor cortex and PMC, anterior and motor cingulate areas, frontal and central operculum, and thalamus	1. Regions of increased FC corresponded with decreased structural connectivity by DTI. 2. Increased FC linked to faster progression rate
Agosta (43)	26 ALS (63), 15 HCs (66)	20	36	SRFC, combined with DTI	1. Increased FC between left primary sensorimotor cortex (ROI) and the right cingulated cortex, parahippocampal gyrus, and cerebellum-crus?. 2. No right primary sensorimotor cortex FC changes were found	1. Patients with no CST abnormalities by DTI had more widespread increased FC to left primary sensorimotor cortex. 2. There was a positive correlations between ALSFRS-R score and increased FC
Tedeschi (44)	20 ALS (60.7), 20 HCs (62.1)	1–168	34.2	ICA, combined with VBM	1. SMN: suppressed RS-fMRI fluctuations in bilateral M1. 2. R-FPN: suppressed RS-fMRI fluctuations in superior frontal gyrus and supramarginal gyrus. 3. DMN showed no significant group difference	1. DMN (specifically PCC) and R-FPN network showed a significant age-by-disease interaction. 2. The volume of gray matter adjacent to regions of reduced FC was decreased
Luo (45)	20 ALS (45.3), 20 HCs (47.1)	15.2	31.9	ALFF, combined with VBM	After gray matter correction: 1. increased ALFF in middle frontal lobe and right inferior frontal gyrus. 2. decreased ALFF in visual cortex, fusiform gyrus and right postcentral gyrus	Disease duration was positively correlated with mean ALFF in left middle frontal gyrus, while rate of disease progression was negatively correlated with it
Tietz (46)	40 ALS (not given), 40 HCs (age matched)	NA	NA	ICA	Increased DMN in frontal and temporal regions	–
Machts (2012 and 2013) (47, 48)	81 ALS (not given), 68 HCs (not given)	NA	NA	SRFC and fALFF	1. SRFC: increased FC of right M1 (ROI) with SMA, precentral and postcentral gyrus; decreased FC of right M1 with PCC, frontal pole, lateral parietal cortex and inferior temporal cortex; No significant differences were found for FC with left M1. 2. fALFF: higher fALFF in right M1 and lower fALFF in PMC	1. FC of both M1 toward contralateral precentral gyri correlated positively with patients’ disease severity as well as fALFF in bilateral PMC. 2. There was an inverse correlation between patients’ ALSFRS-R scores and fALFF in cerebellum
Zhou (49)	12 ALS (49.5), 12 HCs (age matched)	14.2	35.8	Graph theory	18 key nodes were chosen to compare within-motor network FC, ALS patients showed altered pairwise FC in 11 node pairs, both decreased and increased	Increased FC between bilateral superior parietal lobule and right anterior inferior cerebellum related to more severe disease
Fekete (50)	40 MND (36 ALS, 4 PLS, 55), 30 HCs (50)	51	34	SRFC and complex network analysis	1. SRFC: widespread FC alterations in motor network, including regions not obviously clinically affected, such as cerebellum and basal ganglia. 2. Complex network analysis: (1) reduced connectivity of both cortical and subcortical motor areas with non-motor areas, (2) reduced subcortical–cortical motor connectivity and (3) increased connectivity within subcortical motor networks	–
Agosta (51)	20 ALS (61), 15 HCs (63)	29	33	ICA	1. DMN: enhanced connectivity of left precuneus, decreased connectivity of right inferior orbitofrontal gyrus. 2. R-FPN: increased connectivity of right angular gyrus, decreased connectivity of left anterior insula/inferior frontal cortex. 3. L-FPN: increased connectivity of left inferior parietal lobule and left middle cingulum. 4. No change was found in EXN and SLN connectivity	Enhanced parietal connectivity was associated with clinical and cognitive deficits of the patients
Casseb (52)	30 ALS (not given), 24 HCs (not given)	NA	NA	SRFC	BA 4 as ROI, there were no significant results	–
Agosta (53)	24 PLS (62.8), 26 HCs (63.5)	102	36.7	Tractography-derived FC analysis, combined with DTI	1. SMN: increased FC in bilateral precentral and postcentral gyri. 2. Frontal network: increased FC in bilateral ACC and superior medial frontal gyrus, left SMA, and right insula. 3. L-FPN: increased FC in left middle orbitofrontal, inferior frontal, and superior temporal gyri. 4. No FC difference was found in DMN and R-FPN	1. Increased FC within SMN was associated with lower ALSFRS-R scores and more rapid disease progression rate. 2. Increased FC within frontal network was associated with executive dysfunction. 3. Higher FC correlated with greater structural damage to network-specific white matter tracts
Roll (54)	36 ALS (not given), 34 HCs (not given)	NA	NA	SRFC, combined with DTI	Increased FC in all networks except reference network	Disease patterns observed by DTI correlated with increased FC in intrinsic networks
Loewe (55)	64 ALS (not given), 38 HCs (age matched)	NA	NA	Graph theory	1. Increased connectivity in mostly short-range connections within frontal, parietal, occipital, and temporal regions. 2. Decreased FC in motor-related areas include bilateral pre- and postcentral gyrus; decreased temporo-occipital connectivity spread from medial and inferior temporal lobes up to middle occipital lobes	–
Heimrath (56)	9 ALS (57.3), 11 HCs (67.5)	60.2 (since diagnosis)	31.7	Complex network analysis, combined with DTI	Increased FC in parahippocampal and parietal areas of DMN	Increased FC was correlated with pronounced cognitive deficits
Schmidt (57)	64 ALS (56.9), 27 HCs (57.7)	16.5	40	Complex Network Analysis, combined with fiber assignment by continuous tracking	1. Most structurally affected connections overlap with most functionally impaired connections.2.Direct connections of motor cortex are both structurally and functionally more affected than connections at greater topological distance from the motor cortex	
Zhou (58)	12 ALS (49.5), 12 HCs (age matched)	14.2	35.8	ReHo	1. Higher ReHo in S1 (left postcentral gyrus), PMC (right middle frontal gyrus), and sensory association cortex (including bilateral inferior parietal lobule). 2. Lower ReHo in M1 and PMC (right precentral gyrus/superior frontal gyrus), PMC (including left SMA, left precentral gyrus, right superior frontal gyrus), and S1 (right postcentral gyrus)	1. Decreased ReHo in right S1/M1/superior frontal gyrus was correlated with lower ALSFRS-R scores.2.ReHo in left S1 and inferior parietal cortex was negatively correlated with disease duration. 3. Increased ReHo in left S1 corresponds to fast disease progression rate
Zhou (59)	20 ALS (56.9), 20 HCs (57.7)	16.2	35.4	VMHC and SRFC, combined with DTI	1. VMHC: higher VMHC coefficients in SMA, superior frontal gyrus and middle occipital gyrus, lower VMHV coefficients in M1, S1, inferior parietal lobule, cuneus/precuneus and ACC. 2. Significant FC alterations were detected in M1 and frontal/temporal/occipital bole using SRFC based on regions showing abnormal VMHC coefficients	There was a significant positive correlation between VMHC coefficients of M1 and ALSFRS-R scores
Meoded (60)	16 PLS (59.7), 14 HCs (51.6)	104	35.8	Graph theory, combined with probabilistic fiber tracking	12 regions with increased FC with a predominance of cerebrocerebellar connections, strongest between cerebellum and cortical motor areas and between cerebellum and frontal and temporal cortex	Fiber tracking detected no difference in connections between regions with increased FC
Trojsi (61)	15 ALS (61.8), 15 bvFTD (61.5), 15 HCs (62.7)	24	35.6	ICA, combined with DTI and VBM	Decreased RS-fMRI signals within SMN (M1), DMN (PCC), R-FPN (right supramarginal gyrus), EXN (left middle frontal cortex), SLN (medial prefrontal cortex and insula)	ALS and bvFTD share common RS-fMRI connectivity patterns, but differ in DMN, with RS-fMRI signals in PCC enhanced in bvFTD and suppressed in ALS
Buchanan (62)	30 ALS (58.3), 30 HCs (58.5)	24	38.8	Complex network analysis, combined with TBSS	Impaired motor-frontal-subcortical subnetwork involving 4 nodes within M1 (bilateral precentral and paracentral), left superior frontal, left-posterior cingulate and 4 subcortical areas (bilateral pallidum, left thalamus, left caudate)	1. impaired network connections correlated with disease progression rate. 2. Affected network corresponded to impairment of white matter tracts identified by TBSS

*ACC, anterior cingulate cortex; ALFF, amplitude of low-frequency fluctuation; ALS, amyotrophic lateral sclerosis; ALSFRS-R, revised ALS functional rating scale; bvFTD, behavioral variant frontotemporal dementia; DLPFC, dorsolateral prefrontal cortex; DMN, default-mode network; DTI, diffusion tensor imaging; EXN, executive network; fALFF, fractional ALFF; FC, functional connectivity; FPN, frontoparietal network; HC, healthy control; ICA, independent component analysis; L-FPN, left FPN; M1, primary motor cortex; MND, motor neuron disease; NA, not available; PCC, posterior cingulate cortex; PMA, premotor cortex; PLS, primary lateral sclerosis; ReHo, regional homogeneity; R-FPN, right FPN; ROI, region of interest; RS-fMRI, resting state-fMRI; S1, primary sensor cortex; SBM, surface-based morpometry; SLN, salience network; SMA, supplementary motor area; SMN, sensorimotor network; SRFC, seed region-based FC; TBBS, tract-based spatial statistics; VBM, voxel-based morphometry; VMHC, voxel-mirrored homotopic connectivity*.

### Details of MND Patients

In total, 1124 participants with MND were reported, including 1062 ALS, 18 PMA and 44 PLS, although there was considerable overlap between studies. The reported mean disease duration of 35 studies was 28 months (ranging from 14 to 104). The reported mean ALSFRS-R scores of 36 studies was 36.5 (ranging from 27.4 to 41.5). Only nine studies reported that patients were taking riluzole during the examination (17, 19, 22, 33, 40, 43, 44, 51, 56). The El Escorial or Revised El Escorial was commonly used as the standardized diagnostic measure. All studies included a comparison between MND patient group and HC group; besides, five of the studies included an additional comparison group, including patients with peripheral lesions, Kennedy syndrome, tetraplegia, LMN affection and FTD (15, 17, 27, 38, 61).

### Task-Associated fMRI Studies in MND Patients

### Task Design

The experimental paradigms can be divided into block design and event-related (ER) design according to the presentation of the stimuli. Traditional fMRI experiments usually adopt a block design, which consists of two different experimental conditions, namely on (task block) and off (control block) conditions. These two conditions appear alternately in the form of square wave. Signal increases with repeated stimulation due to overlap of “on” and “off” conditions. Block design has the advantage of producing large signal change and thus high statistical power. However, its accumulated effect may produce a large number of false signals and cause a fatigue effect on the participants psychologically. In 1996, Buckner came up with the concept of ER design, or single trial design. In this method, stimulation signals appear as pulses, and there is a time interval between each signal. ER design has a better temporal information regarding response to individual trials and analysis can be restricted to *post hoc* categorization of responses, but it may yield insufficient signal to noise and thus loss of statistical power. In comparison, block design is preferred for detection of activation and ER design is preferred for estimation of hemodynamic response function. In this systematic review, one motor task-associated study (16) and four extra-motor task-associated studies (27, 30, 32, 34) utilized an ER design, and all other studies used a block design.

#### Motor Paradigms

Twelve studies (8–13, 15, 17, 19, 21, 22, 25) utilized the task of limb movement execution, usually the right hand, including finger flexion, finger tapping, button presses, moving a joystick and squeezing a ball (Table 1). In comparison with HCs, ALS patients have shown enhanced bihemispheric activation in the primary motor cortex (M1), premotor cortex (PMC), supplementary motor areas (SMA) and cingulate areas (8–11, 13, 18, 19, 21, 22, 25), more activated in cerebral regions involved in motor learning (cerebellum, brain stem and basal ganglia, especially putamen) (9, 10, 12, 18), and increased recruitment of extra-motor areas [temporal and parietal cortices, primary sensor cortex (S1)] (8, 17, 21, 22, 25). A pattern change and an activity shift to more anterior premotor areas in MND were also observed (8, 13, 21). Furthermore, within the primary sensorimotor cortices there was an alteration in somatotopy, but only in ALS patients with both UMN and LMN affected (11), or UMN only (14). One study compared the activation pattern within the ALS group and found that patients with greater UMN involvement had more robust activation in the anterior cingulate cortex (ACC) and right caudate nucleus than patients with greater LMN involvement. Three studies found reduced activation during motor tasks in MND compared to HCs, and these areas mostly centered on the contralateral M1, SMA, parietal, and prefrontal cortex (9, 12, 15).

Findings from four studies (18, 19, 21, 22) supported that altered brain activation during motor tasks correlated with the amount of UMN involvement. Two studies revealed that when physical impairments got worse in ALS, the activity in the contralateral M1 decreased accordingly (18, 19). In another study, the hypoactivated areas matched with foci of cortical atrophy demonstrated by VBM studies in ALS patients, mainly in primary sensory motor cortex and frontal dorsal PMC (21). At last, patients with faster disease progression had lower activation of extra-motor areas during motor tasks over 1 year than patients with slower disease progression (22).

A different pattern of cortical activity during limb movement imagery or observation was seen in ALS compared to motor execution tasks, though these tasks involve similar areas. Four studies adopted the task of right-hand movement imagery (13, 15, 20, 22). Compared to HCs, ALS patients had increased activity within the M1 and PMC, and decreased activity within the subcortical (e.g., putamen and ACC) and cortical (e.g., hippocampus, left inferior parietal, and medial prefrontal cortex.) structures related to motor memory. Four studies adopted the task of movement observation but revealed contradictory results: two of which found hyperactivation in the PMC and inferior parietal gyrus (24, 25), and the other two studies found hypoactivation in the PMC and inferior frontal gyrus (20, 23).

During tongue movements, the cortical activation pattern in ALS patients without bulbar signs did not differ from HCs. Three studies comparing the activation patterns of ALS patients with bulbar signs and HCs found consistently reduced activity in the M1 and S1 (16, 17, 19). Furthermore, ALS patients without dysphagia showed increased activity in bilateral precentral and postcentral gyri than patients with dysphagia, with additional activation in left thalamus (16). Also, ALSFRS-R score was positively correlated with the signal change in tongue area during tongue movements (19).

#### Extra-Motor Paradigms

Seven studies (26, 31, 32, 34–37) employed language or executive tasks (Table 2). In two studies (26, 37) using letter fluency tasks, it was found that impaired activation in the middle and inferior frontal gyri, ACC, and parietal and temporal cortices was associated with reduced verbal fluency in MND. Inhibitory control, which is the core of execution, has been investigated in five studies (31, 32, 34–36), including Stroop and negative priming tasks, antisaccade and prosaccade tasks, go-stop tasks, go/no-go tasks, and reversal learning tasks. An increased cerebral activation accompanying the performance of the Stroop effect and reduced activation during the negative priming comparison was observed, with most of the altered areas on the left hemisphere (31). The study adopting antisaccade and prosaccade trials revealed enhanced activation in supplementary eye fields and frontal eye fields, and reductions in dorsolateral prefrontal cortex (DLPFC) activity (34). When performing go-stop tasks, patients with ALS had stronger inhibition-related activity in the frontal gyrus, putamen, and pallidum, and stronger execution-related activity in the contralateral sensorimotor cortex (35). In another study, patients’ motor activation was higher than HCs when performed go/no-go tasks during the initial measurement, and declined during the 3-month interval; in contrast, the patients’ novelty-evoked hippocampal activity increased across 3 months (36). At last, the orbitofrontal activity of an ALS patient with impairment on neuropsychological tests and behavioral disturbance was more bilateral and more spatially extensive than controls when performing a probabilistic reversal learning task, while the other patient without behavioral dysfunction did not show these alterations (32).

Three studies (27, 29, 33) investigated the processing of socioemotional stimuli in patients with ALS who do not have dementia, adopting the tasks of receiving socioemotional stimuli from the International Affective Picture System, emotional attribution tasks and identifying the emotional faces as the “target” one, respectively. In ALS, areas with hyperactivity compared to HCs included the right supramarginal area, ACC and bilateral DLPFC (27, 33), and these differences in processing of social information increased over the course of 6 months. The activity in the right-sided frontal areas during processing of aversive emotional stimuli was reduced (29). Another study utilizing a socioeconomic game called “trust task,” which elicited specific patterns of activation along the frontal structure and cingulate gyrus, revealed abnormal alterations of neural activity in the cingulate (28). Finally, in a paradigm where visual, auditory, and somatosensory stimuli were involved, patients with ALS demonstrated decreased response in the secondary visual, auditory and sensory integration areas, and structural damage of white matter in associative cortices were also observed through DTI (30).

### RS-fMRI Studies in MND Patients

Since it is difficult for patients with motor deficits to conduct the execution of certain tasks, RS-fMRI attracts more and more attention in the imaging studies of MND, due to its advantages of time-saving and insusceptibility to cognitive or motor impairments of patients. This method is particularly suitable for the exploration of the systematic damage in patients’ brain network. Details of included studies were listed in Table 3.

#### Methods of RS-fMRI Analysis

With emerging research on resting state network in MND, new analysis techniques are being explored and discovered. Here we provide a brief overview of each analytic method used in the reviewed studies.

Functional connectivity (FC) is evaluated as a measure of the temporal coherence in the functional signal across different regions of the brain. This is generally achieved through seed region-based FC (SRFC) or independent component analysis (ICA). In SRFC, also called ROI analysis, a specific anatomical ROI is selected as the seed region and then a FC map is depicted by detecting temporal correlation between that seed and all other areas in the brain, but the network activity not associated with the ROI will not be detected; thus, this approach might introduce potential biases in evaluation and does not allow a global view of the brain connectivity (63). On the other hand, the ICA approach is more complex as it evaluates the entire brain and decomposes it into multiple independent components, each depicted as a functional map (64). ICA is a data-driven approach without any predefined ROI. Slightly different from ICA, a structural imaging-derived network-guided component analysis was proposed by Douaud et al. (42). Combined with DTI, this tractography-derived FC analysis method allows the integration of structural and FC information.

Graph theory and complex network analysis, which is derived from the former, are a mathematical representation of a real-world complex system, modeled as a set of discrete regions or nodes linked by edges (65). Nodes in large-scale brain networks usually represent specific brain regions, while edges or links represent anatomical, functional or effective connectedness. Unlike graph theory, complex network analysis primarily deals with real-life networks that are larger and more complex, which has been used to detect functional integration and segregation, quantify centrality of individual brain regions or pathways, characterize patterns of local anatomical circuitry, and test resilience of networks to impairments (66).

Amplitude of low-frequency fluctuations (ALFF) measures the intensity of spontaneous changes within the lower frequency range of the BOLD signals (0.01–0.08 Hz), and the power spectrum of BOLD signals is used to calculate correlations to estimate the degree of FC among voxels (67). However, it has been indicated that ALFF is sensitive to the physiological noise, so a fractional ALFF (fALFF) approach is proposed, in which the power spectrum of low frequency is normalized by that of the entire frequency range before statistical comparisons (68). The fALFF method improves the sensitivity and specificity in detecting spontaneous brain activities.

Regional homogeneity (ReHo) is also a data-driven approach that uses Kendall’s Correlation Coefficient to measure the similarity of the time series of a given voxel to those of adjacent voxels in a voxelwise way, and thus a temporal activation map is achieved (69). Greater ReHo values indicate greater FC. However, ReHo depends on the synchronous activities of neighboring voxels, limiting its usage only to study functional connections among anatomically adjacent areas.

Voxel-mirrored homotopic connectivity (VMHC) is a computer-based procedure that quantifies FC between each voxel in one hemisphere and its mirrored counterpart on the other hemisphere, based on the theory that the endogenous spontaneous activity of neurons derived from identical sites on opposite sides of the brain has a high similarity, namely homotopy function.

#### Network and Connectivity Changes

Five studies used the SRFC method, alone or in combination, and several brain areas were chosen as the ROI: primary sensorimotor cortex, M1 or Brodmann (BA) 4 area, SMA, basal ganglia, and cerebellum (43, 47, 50, 52, 54). In the study of Agosta et al. (43), increased FC between the left primary sensorimotor cortex and the right cingulated cortex, parahippocampal gyrus, and cerebellum-crus II were observed; no FC changes of the right primary sensorimotor cortex were found. Using M1 or BA 4 area as a seed, two studies (47, 52) yielded different results: Machts et al. found increased FC of the right M1 with the SMA, precentral, and postcentral gyrus, and decreased FC of the right M1 with the posterior cingulate cortex (PCC), frontal pole, lateral parietal cortex and inferior temporal cortex; while there were no significant results in the study of Casseb et al. Fekete et al. (50) found widespread FC alterations in motor network, including regions not obviously clinically affected, such as the cerebellum and basal ganglia. The study of Roll et al. (54) revealed enhanced FC in all networks except for the reference network and that the hyperconnectivity pattern extended spatially into adjacent brain structures toward more frontal portions.

The ICA approach and its modified version were used in seven studies (38, 41, 42, 44, 46, 51, 61), and network with altered FC in ALS compared to HCs primarily included the default-mode network (DMN), the sensorimotor network (SMN), and the frontoparietal network (FPN). Distinct differences of the DMN were found when comparing ALS patients with HCs, with increased FC in two studies (frontal and temporal regions, left precuneus) (46, 51), decreased FC in three studies (38, 51, 61) (ventral ACC, PCC, bilateral inferior parietal cortex patients, and right inferior orbitofrontal gyrus) and no FC changes in one study (44). Four studies demonstrated reduced FC in the SMN (38, 41, 44, 61), and the alterations mainly centered on the M1 and PMC, while one study revealed increase of FC in the primary sensorimotor cortex and PMC (42). As for the right FPN (R-FPN), suppressed RS-fMRI signals in the superior frontal gyrus and supramarginal gyrus, left anterior insula/inferior frontal cortex were found in three studies (44, 51, 61), and increased connectivity of the right angular gyrus was observed in one study (51). One study reported increased FC of the left inferior parietal lobule and left middle cingulum in the left RPN (L-RPN) (51). One study demonstrated alterations in RS-fMRI signals of the executive network (left middle frontal cortex) and salience network (medial prefrontal cortex and insula) (61), while another study found no significant changes in these two networks (51). One study compared the resting state network of HCs and patients with PLS using ICA, and the results showed increased FC in the SMN, L-FPN, and the frontal network, and no significant alterations were found in the DMN and R-FPN (53).

Brain network topology was assessed through graph theoretical approaches or complex network analysis in nine studies (39, 40, 49, 50, 55–57, 60, 62). Seven studies revealed findings on motor cortex, motor network or motor-related areas, focusing on different aspects (39, 40, 49, 50, 55, 57, 62). Loewe et al. (55) found decreased FC in motor-related areas include bilateral pre- and postcentral gyrus; Zhou et al. (49) found that ALS patients showed altered pairwise FC in 11 out of 18 node pairs chosen from the motor network, both decreased and increased. Further findings from Schmidt et al. (57) indicated that direct connections of motor cortex are both structurally and functionally more affected than connections at greater topological distance from the motor cortex. Fekete et al. (50) discovered reduced subcortical–cortical motor connectivity and increased connectivity within the subcortical motor networks and Buchanan et al. (62) also provided evidence that the motor-frontal-subcortical subnetwork of ALS patients was impaired, involving four nodes within the M1, left superior frontal, left-posterior cingulate and four subcortical areas (bilateral pallidum, left thalamus, left caudate). In the study of Jelsone-Swain et al. (39), overall systemic decrease in FC between the right and left motor cortices in ALS with limb-onset was observed, with a pronounced disconnection between dorsal ROI pairs of the M1. Only the study of Verstraete et al. (40) revealed unchanged functional organization of the motor network. Two studies demonstrated altered connectivity in regions other than the motor network. Heimrath et al. (56) discovered increased FC in the parahippocampal and parietal areas of DMN, and Meoded et al. (60) found 12 regions with increased FC with a predominance of cerebrocerebellar connections.

Two studies using the analysis of ALFF or fALFF showed that, compared with the controls, the ALS patients demonstrated significantly decreased ALFF values in the visual cortex, fusiform gyri and right postcentral gyrus, and significantly increased ALFF values in the left medial frontal gyrus and right inferior frontal areas after gray matter correction (45); and that alterations of motor FC in ALS coincided with altered local fluctuation amplitudes in the M1 and cerebellum independent of the clinical severity (48).

Only one study from the available literature used the analysis of ReHo and one study used VMHC. Researchers found region-dependent ReHo value changes (increase and decrease) in the region of the S1, M1, and PMC (58). And higher VMHC coefficients in the SMA, superior frontal gyrus, and middle occipital gyrus, lower VMHV coefficients in M1, S1, inferior parietal lobule, cuneus/precuneus, and ACC were discovered (59).

### Voxelwise Meta-Analysis

**Figure 2:**
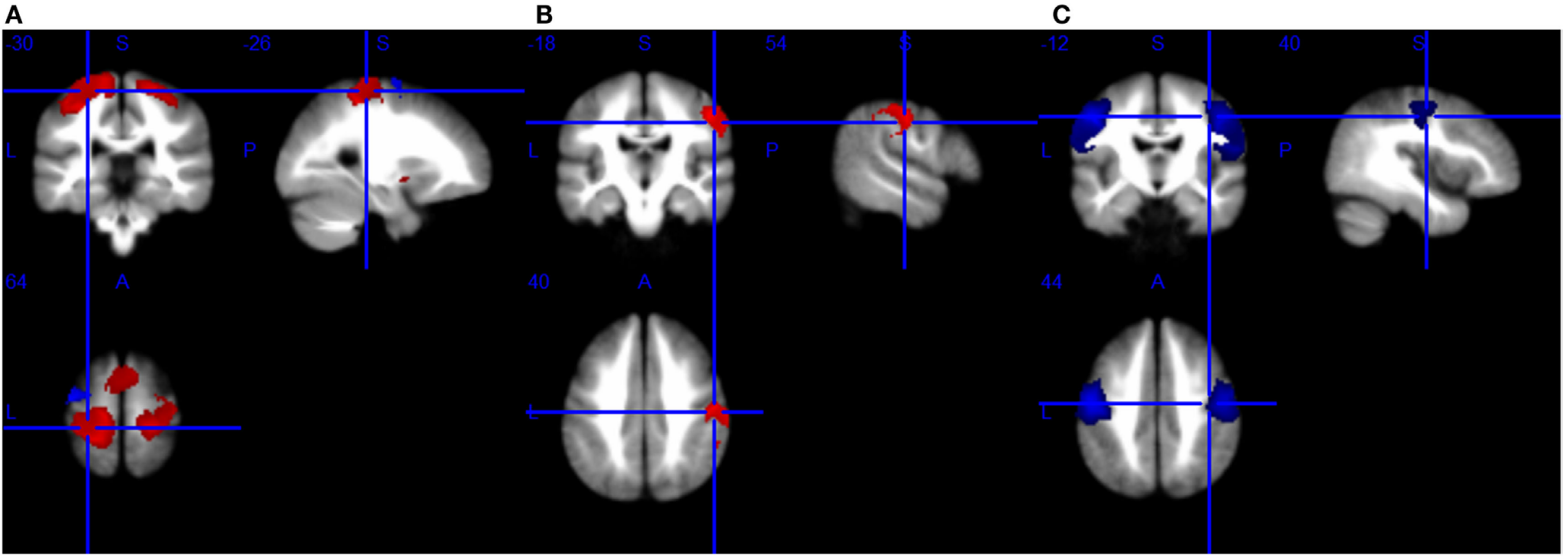
**Brain response abnormalities in motor neuron disease (MND) compared with healthy controls (HCs)**. Areas with increased activity relative to controls are displayed in red, and areas with decreased activity are displayed in blue. In the meta-analyses, compared with HCs, patients with MND had significant hyperactivity in the left postcentral gyrus and right precentral gyrus, and hypoactivity in the left precentral gyrus during right-hand movement execution **(A)**; hyperactivity in the right postcentral gyrus during right-hand movement imagery or observation **(B)**; and hypoactivity in the bilateral precentral gyrus at rest **(C)**.

A total of 30 studies provided coordinates where significant differences between patients with MND and controls were identified. The number of studies employing tongue movement task was too small to conduct a voxelwise meta-analysis. Ten limb movement task-associated studies were divided into right-hand movement execution and right-hand movement imagery or observation. Among the nine extra-motor task-associated studies, only two studies adopted the same task, the letter fluency task, but still, the number of the coordinates was too small to combine. The comparisons in two of the eight included RS-fMRI studies were not made at the whole-brain level, so they were ruled out. Technique details of the remaining 16 studies are presented in Table 4. The main results were illustrated in Figure 2.

**Table 4:** **Technique details of studies included into the voxelwise meta-analysis**.

Study	Scanner (T)	Software	Thickness (mm)	FHWH (mm)	Threshold	Coordinates
Right-hand movement execution						64
Tessitore (12)	1.5	Brain Voyager QX	6	8	*P* < 0.001 (uncorrected)	5
Konrad (10)	1.5	SPM 99	1.2	10	*P* < 0.001 (uncorrected)	6
Stanton (15)	1.5	AFNI	7	7	NA	2
Mohammad (18)	3	Brain Voyager QX	3	8	*P* < 0.05 (uncorrected)	6
Kollewe (19)	3	Brain Voyager QX	3	8	*P* < 0.01 (FDR corrected)	3
Cosottini (21)	1.5	FSL	5	5	*P* < 0.001 (uncorrected)	30
Poujois (22)	1.5	SPM 2	4	8	*P* < 0.05 (corrected for multiple comparisons)	5
Jelsone-Swain (25)	3	SPM 8	3	5	*P* < 0.001 (uncorrected)	7
Right-hand movement imagery or observation						46
Lule (13)	1.5	SPM 2	3.5	10	NA	6
Stanton (14)	1.5	AFNI	7	7	NA	2
Poujois (22)	1.5	SPM 2	4	8	*P* < 0.05 (corrected for multiple comparisons)	2
Li (24)	3	SPM 8	4	8	*P* < 0.001 (uncorrected)	5
Jelsone-Swain (25)	3	SPM 8	3	5	*P* < 0.001 (uncorrected)	31
Resting state						43
Mohammad (38)	3	Brain Voyager QX	3	8	*P* < 0.001 (uncorrected)	5
Tedeschi (44)	3	Brain Voyager QX	4	6	*P* < 0.005 (uncorrected)	3
Luo (45)	3	SPM 8	1	8	*P* < 0.05 (FWE corrected)	5
Agosta (51)	1.5	SPM 8	4	6	*P* < 0.001 (uncorrected)	6
Zhou (58)	3	SPM 8	4	6	*P* < 0.05 (corrected for multiple comparisons)	9
Agosta (53)	3	FSL	2.5	6	*P* < 0.05 (FWE corrected)	15

*AFNI, analysis of functional neuroimages; FDR, false discovery rate; FWE, family-wise error; FHWH, full-width half maximum; FSL, FMRIB Software Library; NA, not available; SPM, Statistical Parametric Mapping; T, Tesla*.

#### Changes in Brain Response to Right-Hand Movement Execution

As shown in Table 5 and Figure 2A, compared with HCs, patients with ALS had hyperactivity in the left postcentral gyrus (BA 3), corpus callosum, right precentral gyrus (BA 4) and left striatum, and had hypoactivity in the left precentral gyrus (BA 6) during right-hand movement execution. In Jackknife sensitivity analysis, the software repeated the mean analysis several times, each time including all the studies but one to test the replicability of the result. However, we noted that in the sensitivity analysis, hyperactivity in the corpus callosum was reported in only three studies; therefore, we conservatively did not consider it as a significant finding. We performed a meta-regression analysis for the ALSFRS-R score and found that it was positively associated with activation in the corpus callosum (MNI coordinate: 10, −8, 58, SDM-Z 3.354, *P* = 0.000330269, no. of voxels 170).

**Table 5:** **Brain response abnormalities in ALS compared to HCs in task of right-hand movement execution**.

Region	MNI coordinate (*x*, *y*, *z*)	SDM-Z	*P*-values	Voxels	Clusters of breakdown (no. of voxels)	Jackknife sensitivity analysis
**Hyperactivity in als patients compared to HCs**						
Left postcentral gyrus, BA 3	−34, −38, 54	3.134	0.000010312	1798	L postcentral gyrus (666)	7 out of 8
					L precentral gyrus (337)	
					L paracental lobule (273)	
					L prcuneus (81)	
					L inferior parietal (47)	
					L hand superior U tract (43)	
					L superior parietal gyrus (31)	
					L superior longitudinal fasciculus II (20)	
					Corpus callosum (290)	
					Undefined (10)	
Corpus callosum	10, 6, 52	2.442	0.000190973	1932	R SMA (536)	3 out of 8
					R median cingulate/paracingulate gyri (234)	
					L SMA (678)	
					L median cingulate/paracingulate gyri (329)	
					L superior fontal gyrus, medial (26)	
					L median network, cingulum (10)	
					Corpus callosum (109)	
					Other (10)	
Right precentral gyrus, BA 4	36, −22, 54	3.037	0.000020623	1593	R precentral gyrus (909)	8 out of 8
					R postcentral gyrus (357)	
					R superior frontal gyrus, dorsolateral (74)	
					R middle frontal gyrus (69)	
					R supramarginal gyrus (33)	
					R superior longitudinal fasciculus II (29)	
					R frontal superior longitudinal (16)	
					R paracentral lobule (11)	
					Corpus callosum (73)	
					Other/undefined (22)	
Left striatum	−20, 2, −4	1.895	0.002002418	46	L striatum (31)	5 out of 8
					Other/undefined (15)	
**Hypoactivity in als patients compared to HCs**						
Left precentral gyrus, BA 6	−38, −8, 62	−1.093	0.000087738	750	L precentral gyrus (436)	7 out of 8
					L postcentral gyrus (197)	
					L superior fontal gyrus, dorsolateral (67)	
					Corpus callosum (41)	
					Other/undefined (9)	

*ALS, amyotrophic lateral sclerosis; BA, Brodmann; HC, healthy control; MNI, Montreal Neurological Institute; SDM, signed differential mapping; SMA, supplementary motor area*.

#### Changes in Brain Response to Right-Hand Movement Imagery or Observation

As shown in Table 6 and Figure 2B, compared with HCs, patients with ALS had hyperactivity in the right postcentral gyrus (BA 4), left superior frontal gyrus (dorsolateral, BA 6), and right insula (BA 48), and no hypoactivity was found during right-hand movement imagery or observation in the meta-analysis. Nevertheless, the results changed remarkably in the following Jackknife analysis, with the highest replicability being only three out of five, which meant the robustness, specifically the sensitivity, was not very good. In the meta-regression analysis, ALSFRS-R was negatively associated with activation in the right median cingulate/paracingulate gyri (MNI coordinate: 6, 12, 42, SDM-Z −2.066, *P* = 0.000252903, no. of voxels 556). Considering the high heterogeneity of the included studies, statistical significance of this meta-regression should be taken with caution.

**Table 6:** **Brain response abnormalities in ALS compared to HCs in task of right-hand movement imagery or observation**.

Region	MNI coordinate (*x*, *y*, *z*)	SDM-Z	*P*-values	Voxels	Clusters of breakdown (no. of voxels)	Jackknife sensitivity analysis
**Hyperactivity in ALS patients compared to HCs**						
Right postcentral gyrus, BA 4	48, −20, 44	2.121	0.000185788	719	R postcentral gyrus (357)	3 out of 5
					R suparamarginal gyrus (272)	
					R precentral gyrus (56)	
					R inferior parietal gyri (27)	
					Other (7)	
Left superior frontal gyrus, dorsolateral, BA 6	−22, −6, 56	1.746	0.001243770	73	L superior fontal gyrus, dorsolateral (26)	0 out of 5
					L precentral gyrus (22)	
					Other (25)	
Right insula, BA 48	34, −8, 14	1.684	0.001770139	69	R insula (39)	1 out of 5
					Other/undefined (30)	

*ALS, amyotrophic lateral sclerosis; BA, Brodmann; HC, healthy control; MNI, Montreal Neurological Institute; SDM, signed differential mapping*.

#### Changes in Brain Response at Rest

As shown in Table 7 and Figure 2C, compared with HCs, patients with MND had hypoactivity in the right precentral gyrus (BA 4) and left precentral gyrus (BA 6), and no hyperactivity was found at rest. The whole brain Jackknife sensitivity analysis indicated activation reductions in precentral gyrus on both sides were moderately replicable because they were preserved four out of the six combinations. The meta-regression analysis showed ALSFRS-R score was negatively associated with activation in the right angular gyrus (MNI coordinate: 54, −52, 30, SDM-Z −2.6, *P* = 0.000629604, no. of voxels 1127).

**Table 7:** **Brain response abnormalities in ALS compared to HCs at rest**.

Region	MNI coordinate (*x*, *y*, *z*)	SDM-Z	*P*-values	Voxels	Clusters of breakdown (no. of voxels)	Jackknife sensitivity analysis
**Hypoactivity in ALS patients with bulbar sign compared to HCs**						
Right precentral gyrus, BA 4	42, −20, 64	−1.478	0.001728892	41	R precentral gyurs (36)	4 out of 6
					R postcentral gyrus (5)	
Left precentral gyrus, BA 6	−38, −14, 56	−1.337	0.003860295	42	L precentral gyurs (32)	4 out of 6
					L postcentral gyrus (10)	

*ALS, amyotrophic lateral sclerosis; BA, Brodmann; HC, healthy control; MNI, Montreal Neurological Institute; SDM, signed differential mapping*.

## Discussion

The use of fMRI in MND research is relatively new; however, the number of published studies grows steadily with the ultimate goals of improved understanding of the underlying pathophysiologic mechanism of this disorder as well as the identification of biomarkers of MND progression. Thus, the literature has uncovered a wide array of brain regions that exhibit group differences between MND patients and HCs. Despite the variability across included studies with respect to study designs and analytic approaches, a number of consistencies emerged and demonstrated similar findings to the PET literature that has been reviewed elsewhere (70).

### Implications of the Main Findings

#### Motor Paradigms

We observed an enhanced activation in motor areas during movement in patients with ALS, including the bilateral M1, PMC, and SMA in early stages, along with additional recruitment of cerebral regions for higher order motor processing, determined by motor neuron involvement (especially UMN impairments) in the long run. Such alterations might represent a compensatory cortical plasticity, as new synapses and pathways are developed to compensate for the loss of pyramidal cells in the M1 and the reduction of local inhibitory interneuronal function (71). An anterior shift of activity and spread to encompass the sensorimotor cortices and temporoparietal associative sensory, which has also been noted in stroke patients, other neurodegenerative disorders and the aging brain, lend support to this theory (72). The striatal pattern of activation indicate that ALS patients need to recruit basal ganglia system to complete simple finger movement tasks, compared to that this system is normally recruited in more complex motor behaviors in controls. This difference is interpreted as a pattern of functional adaptation to the corticospinal tract dysfunction, suggesting ALS patients may also recruit existing neuronal pathways to compensate for neuronal loss of the primary motor region (72). However, the compensation is not unlimited. A longitudinal fMRI study found two distinct stages of neuroplastic changes when comparing the motor activations in three groups of ALS patients with different degrees of weakness: first, an increase of the activated area in the contralateral sensorimotor cortex irrespective of the degree of weakness; second, reduction of signal change and beta weights with increasing weakness (18). The fact that the size of the activated cluster did not change between the ALS groups suggested the spread of activation due to loss of intracortical inhibition would reach a ceiling early in the disease, and the reduction of signal change and beta weights was a consequence of ongoing loss of UMN (18).

The different activation pattern of movement imagery might be an indication of disruption of the normal networks associated with motor imagery (4). The decrease of activation in ALS patients with bulbar signs when performing voluntary saliva swallow or tongue vertical movements stands in prominent contrast to the increase of activity observed in ALS patients when performing limb movements. Lack of compensatory capacity for bulbar movements compared with spinal movements might be one of the potential mechanisms, indicating fundamental differences in the neurodegenerative and subsequent reorganization processes for limb and bulbar movements (19).

#### Extra-Motor Paradigms

Through extra-motor paradigms, fMRI data provided evidence for a multisystem involvement of cognitive, socioemotional, and sensory processing pathways in patients with MND. Decreased activation in the prefrontal areas related to fluency and confrontation naming deficits in patients with MND regardless of UMN signs has been observed, suggesting an impairment of lexical and phonological processing as well as the dysfunction of working memory (26, 37). In studies investigating inhibitory control of ALS, in spite of the utilization of different kinds of tasks, they consistently revealed that ALS patients had difficulty in achieving response suppression (31, 34, 36). Inhibition of automatic responses is a crucial process within the executive system, and these results suggested a direct link between a particular deficit of cognitive process and a functional impairment in the prefrontal cortex in patients with ALS. The findings that novelty-evoked hippocampal activity in the ALS group during go/no-go task increased across 3 months and that ALS patients’ enhanced functional activity found in specific areas of the saccade network associated with better responses, both reflect the build-up of the compensatory processes typically observed at the beginning of neurological lesions and fit with the notion of functional compensatory plasticity following their cognitive impairments (34, 36).

Further changes in cortical pattern activation were observed in non-demented ALS during the processing of socioemotional stimuli. When they were shown pictures of persons in emotional situations, ALS patients presented an enhanced activity in the supramarginal area on the right side, which is a part of the social information-processing pathway (27). The elevated activity suggests an altered sensitivity to social–emotional cues in ALS patients without significant cognitive impairments. Reduced activity in the right-sided frontal regions during processing of aversive emotional stimuli also corroborates the assumption of dysfunction in emotional processing network in ALS (29). Though there is limited anatomical and clinical evidence for sensory processing deficits in ALS, it seems that the neurodegeneration involves visual, auditory, and somatosensory cortical areas as well (30).

#### Resting State-Functional Magnetic Resonance Imaging

To date, there has been no established consensus about the best way to compare findings from studies that utilize different analytical approaches. Each method that has been described in this review produced, mostly consistent or similar results, yet in a few instances, contradictory findings as well. In general, we detected significant changes in the SMN, DMN, and FPN. The SMN has been linked to motor control and the latter two have been shown to be involved in cognitive and executive processes. The present results once again support impairments in extra-motor system in ALS.

Several studies reported significantly reduced FC within SMN and DMN and significantly overlapping the areas of structural change, whereas other studies have identified regions of increased FC, including somatosensory and extra-motor areas. A possible explanation to the controversial changes is that in the early stage of the disease, when most function is still preserved, FC has already begun to degrade; however, with increased burden of pathology, loss of local inhibitory circuitry starts to manifest, leading to an increase of FC (73). This concept is supported by the evidence that patients with higher disease burden had stronger network connectivity than patients who were less affected by ALS (49). Another assumption interprets the increased FC as a compensation for the structural damage, but this increase will be exhausted with disease progression (71). In agreement with the second hypothesis, ALS patients with preserved corticospinal tract had more widespread connectivity than those with severe damages assessed by DTI (43). The latter hypothesis is further fostered by a significant disease-by-age interaction in the DMN, especially in the PCC, found in the ALS group, where signals correlated with age positively in patients but negatively in controls (44). The authors think that the interaction unravels a possible mechanism of compensation between motor and extra-motor systems, emerging as a supplementary functional push to help motor disturbances (44). The reported discrepancy probably reflects the different compositions of the patient groups, which varied in the degrees of the disease severity and therefore in different pathological stages.

### Implications of the Voxelwise Meta-Analysis

For the first time, we used quantitative SDM meta-analytic methods to synthesize findings from 16 functional neuroimaging studies of MND. Compared with an individual fMRI study, this technique is able to establish consistent fMRI data from the included studies. Results revealed reliable clusters of abnormal activation in MND within the regions comprising precentral and postcentral gyrus on both hemispheres when compared with HCs. Moreover, these altered cerebral activation map changes corresponded to atrophy detected by meta-analysis of VBM studies in ALS (74). We also found that the symptom severity assessed by ALSFRS-R scores was associated with hypoactivity in the corpus callosum during right-hand movement and hyperactivity in the right angular gyrus at resting state.

It is noteworthy that, in the Jackknife analysis, the robustness of some regional activity changes was not very high, in some cases, tasks of right-hand movement imagery or observation for example, even down to 0. Several explanations could account for the instability. First, heterogeneity of the disease process might generate independent impacts on the brain’s functional state, and movement imagery or observation in particular might represent highly heterogeneous conditions since it is very hard to quantify. Second, the heterogeneity of the methodologies in the fMRI studies, including different preprocessing protocols and analytic approaches, might also be a critical factor. Finally, the number of eligible studies was relatively small and findings in the original studies were less likely to overlap at the voxel level to reach sufficient statistical power to demonstrate concordant results.

Meanwhile, the meta-analysis is unable to answer whether the reported changes represent a tendency for the development of ALS or the consequence of the illness; thus, could not provide useful information to verify any particular mechanism. There is a need for further large developmental studies that examine the association between brain function alterations and symptom onset longitudinally to fully answer this question.

### Limitations

The reviewed studies have several limitations. The main limitation lies in the variation of the studied population, which differed largely in disease duration and severity. Another important source of bias may be the effect of medication. Some articles reported their patients of the sample were receiving riluzole while others did not mention; however, riluzole is known to increase intracortical inhibition and therefore interfere with the BOLD signals according to a previous study (75). As for the voxelwise meta-analysis, though it is able to provide an excellent control of false positives, it is difficult to completely avoid false-negative results (6). The breakdown of a cluster should not be understood as “all these areas are abnormal” but as “one or more of these areas are abnormal,” because normal brain regions close to those abnormalities might appear to be abnormal artificially. Furthermore, this approach is based on pooling of summarized coordinates rather than raw statistical brain maps, which may result in less accuracy (6). Meanwhile, the sample size of our included studies is relatively small, which limits the generalization of the results. These limitations, along with the methodological differences, make the interpretation of the final analysis difficult. As recently recommended (76), future studies should include sufficient numbers of MND patients in different disease stages to provide better insight into changes of cerebral function and their relation with the disease process.

In conclusion, these findings are preliminary, sometimes even contradictory, and do not allow a complete and thorough understanding of the functional alterations in MND. However, the current evidence sufficiently suggests that abnormal activation in motor areas including the bilateral M1 and the SMN contribute to the pathophysiology of the illness, which is also verified by the results of SDM meta-analysis and that patients might recruit other regions to compensate the structural damages. We also documented reliable findings that ALS is not confined to the motor system, but is a multisystem disorder involving extra-motor cortex areas, causing dysfunction in cognition and deficits in socioemotional and sensory processing pathways. Future studies will benefit from larger and more homogenous cohorts, and standard models for comparison between different analytic methods. Longitudinal fMRI studies tracking patients from disease onset and continuous follow-up are also worthwhile.

## Conflict of Interest Statement

The authors declare that the research was conducted in the absence of any commercial or financial relationships that could be construed as a potential conflict of interest.
